# Recent increases in Arctic freshwater flux affects Labrador Sea convection and Atlantic overturning circulation

**DOI:** 10.1038/ncomms10525

**Published:** 2016-01-22

**Authors:** Qian Yang, Timothy H. Dixon, Paul G. Myers, Jennifer Bonin, Don Chambers, M. R. van den Broeke, Mads H. Ribergaard, John Mortensen

**Affiliations:** 1 School of Geosciences, University of South Florida, 4202 E Fowler Avenue, Tampa, Florida 33620, USA; 2 Department of Earth and Atmospheric Sciences, University of Alberta, 1-26 ESB, Edmonton, Alta, Canada T6G 2E3; 3 College of Marine Science, University of South Florida, St. Petersburg, Florida 33701, USA; 4 Institute for Marine and Atmospheric Research Utrecht, Utrecht University, P.O. Box 80.005, 3508 TA, Utrecht, The Netherlands; 5 Danish Meteorological Institute, DK-2100 Copenhagen, Denmark; 6 Greenland Climate Research Centre, Greenland Institute of Natural Resources, PO Box 570, 3900 Nuuk, Greenland

## Abstract

The Atlantic Meridional Overturning Circulation (AMOC) is an important component of ocean thermohaline circulation. Melting of Greenland's ice sheet is freshening the North Atlantic; however, whether the augmented freshwater flux is disrupting the AMOC is unclear. Dense Labrador Sea Water (LSW), formed by winter cooling of saline North Atlantic water and subsequent convection, is a key component of the deep southward return flow of the AMOC. Although LSW formation recently decreased, it also reached historically high values in the mid-1990s, making the connection to the freshwater flux unclear. Here we derive a new estimate of the recent freshwater flux from Greenland using updated GRACE satellite data, present new flux estimates for heat and salt from the North Atlantic into the Labrador Sea and explain recent variations in LSW formation. We suggest that changes in LSW can be directly linked to recent freshening, and suggest a possible link to AMOC weakening.

## Abstract (web summary)

Labrador Sea convection and the Atlantic overturning circulation are sensitive to surface freshening of the North Atlantic. Here, the authors show that the recent increases in Arctic freshwater flux can directly weaken Labrador Sea convection and potentially affect the overturning circulation.

## Graphical abstract

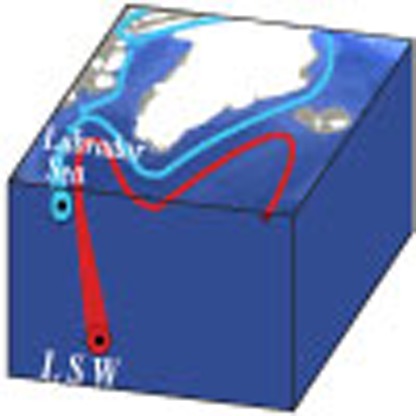

It has long been accepted that the Atlantic Meridional Overturning Circulation (AMOC) has two stable modes^1^,^2^,^3^, and that anthropogenic warming could weaken or shut down the AMOC^4^,^5^. Recent accelerated melting of the Greenland ice sheet is freshening the North Atlantic^6^,^7^,^8^,^9^,^10^. So-called ‘hosing experiments', where freshwater may be distributed over broad or narrow regions of the North Atlantic in numerical models, have been used to study the sensitivity of the AMOC to freshwater flux^11^,^12^,^13^,^14^,^15^,^16^,^17^. Some of these studies suggest that AMOC strength is sensitive to Greenland melting^11^,^17^, while others do not^12^,^14^,^16^. A few studies suggest that freshwater additions of 0.1 Sv (100 mSv)^18^,^19^,^20^ or possibly less^11^,^17^ could affect the AMOC.

Changes in the AMOC are difficult to measure directly: currents that comprise the deeper, southward flowing portions can be diffuse and/or spatially and temporally variable, and instrumental drift can mask subtle, long-term changes in oceanic properties. It is also challenging to separate changes forced by anthropogenic warming from natural variability. The AMOC is difficult to model numerically: model grids may be too coarse to reflect realistic oceanic processes and geographic constraints, and feedbacks among atmosphere, ocean and cryosphere (land and sea ice) are poorly known.

The Labrador Sea is a key location for the formation of one of the dense, deep-water components of the AMOC via winter convection; however, the process is sensitive to surface conditions^21^. Wood *et al*.^5^ suggest the possibility of a shutdown in Labrador Sea convection in response to global warming. Kuhlbrodt *et al*.^22^ provide a theoretical stability analysis, and suggest that winter convection in the Labrador Sea can be turned off by increased freshwater input. Unfortunately, winter convection here is difficult to observe directly because of extreme conditions and its small spatial scale.

Here we consider recent Labrador Sea changes associated with an increased freshwater flux. We derive a new estimate for recent increased freshwater flux into the sub-polar North Atlantic, and suggest that because of the clockwise nature of ocean circulation around Greenland^23^, most of this increase is being focused towards the Labrador Sea (Fig. 1), magnifying its impact and increasing the likelihood of significant effects on the AMOC.

## Results

### Recent accelerated melting of the Greenland ice sheet

Numerous studies have described recent acceleration of Greenland's ice mass loss^6^,^7^,^8^,^9^,^10^. We use GRACE data updated to October 2014 to derive a new acceleration estimate and its onset time (Methods). GRACE data and uncertainty estimates follow Bonin and Chambers^24^. We fit a constant acceleration model to the data, and extrapolate the best-fit model back to the time of zero mass loss rate, obtaining 20-Gt per square year acceleration with a start time of 1996±1.4 years (Fig. 2). Several lines of evidence suggest that the ice sheet was relatively stable from 1980 to the early 1990s (refs 25, 26), and we use that assumption in our modelling of GRACE data and freshwater flux calculations (below and Methods section).

### Irminger Water heat and salt fluxes

**Figure 1:**
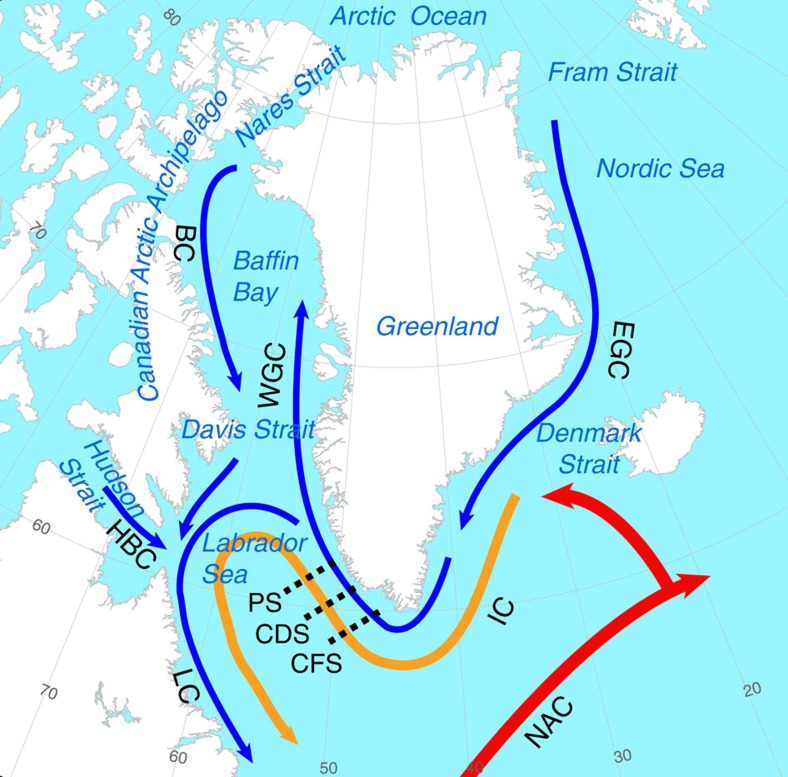
Study region showing oceanographic sections and major currents around Greenland. Red and orange arrows indicate Atlantic-origin water and blue arrows indicate Arctic-origin water. BC, Baffin Current; CDS, Cape Desolation Section; CFS, Cape Farewell Section; EGC, East Greenland Current; HBC is Hudson's Bay Current; IC, Irminger Current; LC, Labrador Current; NAC, North Atlantic Current; PS, Paamiut Section; WGC is West Greenland Current. Three-dimensional structure of major water masses in the Labrador Sea is shown in Supplementary Fig. 1.

Warming of sub-polar mode waters including Irminger Water in the mid- to late-1990s (refs 27, 28) is thought to influence coastal mass loss in Greenland by increasing submarine melting of outlet glaciers and related dynamic effects^29^,^30^,^31^. Here we examine the variability of heat and salt fluxes of Irminger Water along three sections (Fig. 1) offshore southwest coastal Greenland for the period 1949–2013 (Methods). Currents associated with the sub-polar gyre here are quite compact as they round the southern tip of Greenland, limiting spatial variability and facilitating accurate flux measurements because the cross-section area of current is well defined. Note that, while the flux (*sensu stricto*) is flow rate per unit area and transport (or total flux) represents the flux integrated over the larger area of interest, the terms ‘flux' and ‘transport' are often used interchangeably in the oceanographic literature. We follow the broader (*sensu lato*) usage here.

**Figure 2:**
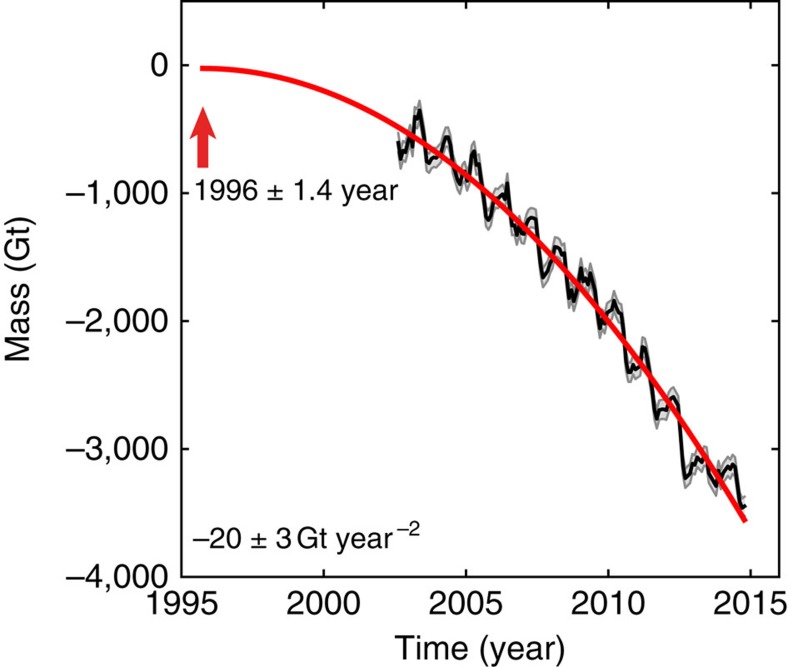
Mass change of Greenland estimated from GRACE for the period 2002–2014. Black curve shows data, grey shading indicates monthly uncertainty and red curve shows the best fitting constant acceleration model. Onset time of acceleration defined when the rate of mass change is zero, in 1996 (red arrow), with mass arbitrarily set to zero.

**Figure 3:**
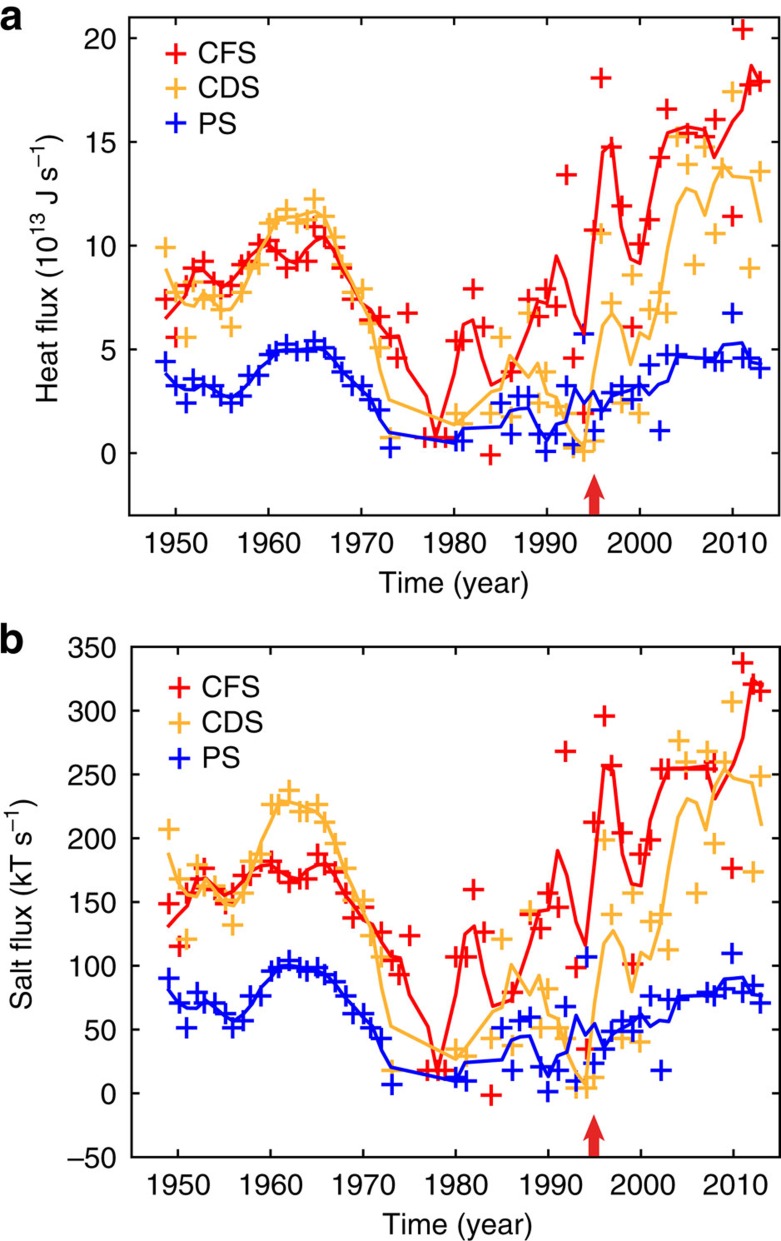
Heat and salt fluxes of Irminger Water for the period 1949–2013. (**a**) Heat and (**b**) salt fluxes of Irminger Water are measured at three sections in southwest Greenland. Locations of three sections are shown in Fig. 1. CDS, Cape Desolation Section; CFS, Cape Farewell Section; PS, Paamiut Section. Solid line represents a 3-year running average, yearly data shown by plus signs. Red arrow marks the onset time of accelerated mass loss for Greenland estimated from GRACE (Fig. 2).

We carry out our analysis on the upper 700 m, the greatest depth common to all years, binned on a 2-m vertical grid. Time series of heat and salt fluxes at the three sections are shown in Fig. 3. At the southernmost Cape Farewell section, both heat and salt fluxes experienced a large multi-year anomaly around 1995, followed by another in the late 1990s. The heat flux was 80% higher than a previous multi-year anomaly in the 1960s. Similar variability is seen at the more northerly Cape Desolation section, although salinities and heat are generally lower, and only exceed previous levels after 2000. No significant anomalies were observed at the northernmost Paamiut section during these times; however, the heat and salt fluxes are still roughly 50% higher after 2000 than they were in the 1980s, and approach levels that are not seen since the 1960s. Thus, we conclude that Irminger Water became warmer and saltier in the mid-late 1990s, which agrees well with the onset time of recent accelerated Greenland mass loss (Fig. 2). This is consistent with the idea that accelerating ice mass loss in the mid-late 1990s reflects, at least in part, the appearance of warmer Irminger Water on the peripheral continental shelf at that time^29^. The anomalous heat flux we observe off southern Greenland in the mid-1990s can be directly tied to warming of the North Atlantic (Supplementary Fig. 2; see also ref. 31).

Northward reduction in heat and salt transport between the Cape Desolation and Paamiut sections likely reflects strong offshore eddy transport^32^, advecting Irminger Water into the interior of the Labrador Sea. However, since the sections are only occupied once a year in summer, some seasonal aliasing is possible. The eddies also transport fresh shelf water into the Labrador Sea^33^.

### Estimates of the freshwater flux into the Labrador Sea

Major sources of freshwater entering the Labrador Sea include precipitation, oceanic transport and melt from the Greenland ice sheet, glaciers in the Canadian Arctic Archipelago (CAA) and Arctic sea ice. Precipitation in the Labrador Sea region is about 20–30 mSv (ref. 34), and there has been a general increase over the North Atlantic region in the last few decades as the hydrologic cycle accelerates^35^. Oceanic transport from the Arctic Ocean is the largest source of Labrador Sea freshwater and is derived from several sources, including the difference between precipitation and evaporation, river discharge and fractionation associated with annual sea ice formation. Peterson *et al*.^36^ show that the average annual river discharge from six rivers in Eurasia into the Arctic Ocean has increased by 7% (∼4 mSv) from 1936 to 1999. The Arctic Ocean exports low-salinity water to the North Atlantic through two main pathways: Fram Strait east of Greenland and the CAA west of Greenland. The CAA pathway has three main routes: Barrow Strait, Nares Strait and Cardigan Strait-Hell Gate. Roughly, 100 mSv of freshwater is exported through each of the east and west pathways, relative to a reference salinity of 34.80 (ref. 37). Within broad error bars, oceanic transport from the Arctic Ocean is relatively stable on the decadal timescale, although there has been some reduction through the CAA and then Davis Strait, and shorter-term fluctuations are common^37^,^38^,^39^.

Here we focus on three Arctic freshwater sources that are undergoing rapid increases, which likely contribute freshwater to the Labrador Sea, and which can be estimated from remote observations: the Greenland ice sheet, CAA glaciers and Arctic sea ice. We also consider snowmelt runoff from tundra in Greenland and the CAA as they follow directly from the same models used to quantify Greenland ice sheet and CAA glacier melt^40^,^41^. As we are not considering the large Arctic oceanic transport term and several other sources, our estimate is a minimum estimate.

**Figure 4:**
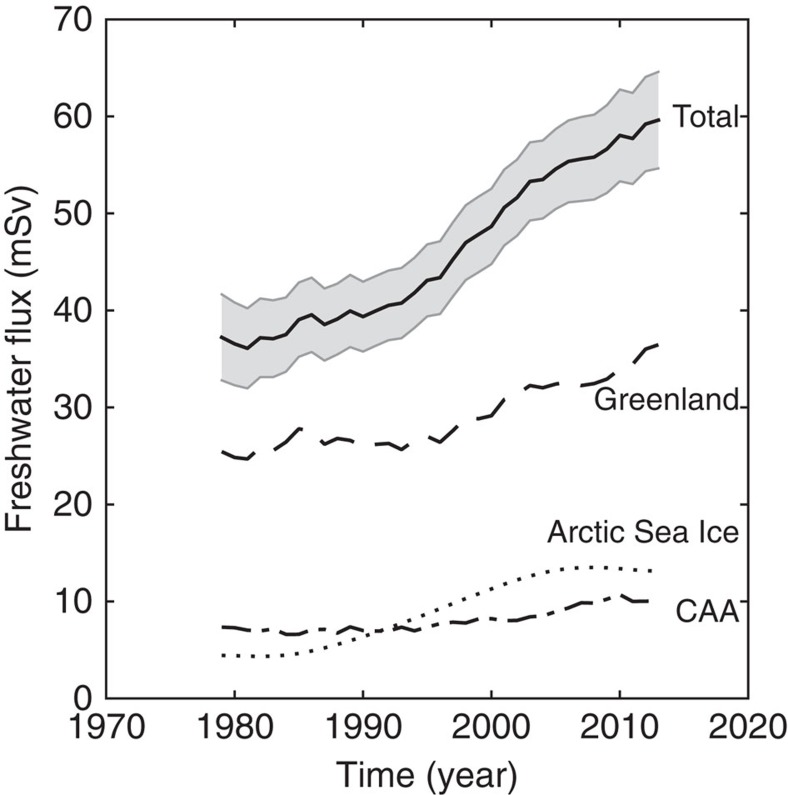
Freshwater flux from Greenland and CAA and Arctic sea ice for the period 1979–2013. For Arctic sea ice, we plot only changes in flux (see text). The sum of these sources (Total) is also plotted. Grey shading indicates propagated uncertainty (see Supplementary Note 1).

The freshwater flux from Greenland is composed of ice and tundra runoff plus ice discharge; this quantity is equal to accumulation minus mass balance (Methods). We derive mass balance for Greenland from GRACE, while accumulation is obtained from the RACMO2.3 model^42^,^43^. Our GRACE data suggest that mass loss of the Greenland ice sheet accelerates from 1996 onwards (Fig. 2; Methods). Our mass balance estimate agrees with the estimate of Box and Colgan^26^, with the Greenland ice sheet in near balance from 1980 to about 1996, after which it starts to lose mass (Supplementary Fig. 3). Therefore, we assume that between 1980 and 1996 the freshwater flux from Greenland is approximately equal to accumulation; after 1996, the freshwater flux from Greenland equals the sum of mass loss and accumulation (Supplementary Fig. 3). Since the accumulation is highly variable from year to year, we smooth it with a 5-year running mean. Figure 4 shows the resulting freshwater flux estimates from Greenland. This approach yields freshwater flux estimates that agree with those of Bamber *et al*.^40^ during the period of data overlap, once a correction for solid ice discharge is applied^8^ (Supplementary Fig. 4). Freshwater from the CAA is approximated by ice and tundra runoff predicted by RACMO2.3 since ice discharge (0.16 mSv) is negligible^44^.

Large amounts of Arctic sea ice and freshwater are exported from the Arctic Ocean to the North Atlantic through several pathways. Of these, Fram Strait and the CAA are the major ones; nearly all (∼98%) Arctic Ocean exports drain through them^37^. However, there are large uncertainties in these fluxes^37^. We focus on changes in the freshwater flux as inferred from recent accelerated melting of Arctic sea ice, assuming that the change is partitioned the same way as the total export, that is, 98% of the change is advected through Fram Strait and the CAA. Changes in the annual minimum of Arctic sea ice volume are a relevant indicator (see Methods and Supplementary Methods). We first use the annual minimum volume predicted by the Pan-Arctic Ice Ocean Modeling and Assimilation System (PIOMAS) model^45^. We also apply the same method to the Arctic sea ice extent and sea ice area data sets^46^, where ‘extent' defines a region as either ‘ice-covered' or ‘not ice-covered' using a threshold of 15%; ‘area' is a more conservative estimate, defined as the percentage of actual sea ice within a given data cell. We assume a standard ice thickness of 2 m (ref. 47) to convert ice extent and ice area to volume, obtaining results that are somewhat smaller than the PIOMAS volume model. Figure 4 shows results from the PIOMAS volume model. Results from the other two approaches are shown in Supplementary Figs 5 and 6.

Figure 4 also shows the summed result from these various freshwater sources (recall that this summed value does not include several major sources and is therefore a minimum estimate), which is our estimate of the freshwater flux into the sub-polar North Atlantic. The freshwater flux from Greenland is relatively stable from 1979 to the mid-late 1990s and then increases. The freshwater flux from the CAA is relatively stable until the early 2000s and then increases. Freshwater flux from Arctic sea ice increases mainly during the period 1990–2000. The total freshwater flux for the sub-polar North Atlantic from these sources is about 60 mSv by 2013, with an increase of 20 mSv during the last two decades. Of this, ∼12 mSv comes from the Greenland ice sheet and CAA glaciers, whereas ∼8 mSv represents excess melting of Arctic sea ice.

Focused freshwater flux into the Labrador Sea has the potential to disrupt the AMOC by increasing the buoyancy of surface waters and reducing the formation of dense, deep water^13^. How much of the enhanced freshwater flux that we calculate actually winds up in the Labrador Sea?

Myers *et al*.^33^,^48^ showed that a significant fraction of freshwater originating in and around Greenland is transported to the Labrador Sea: melt water from eastern Greenland is entrained in the East Greenland Current, where it moves south and merges with the Irminger Current as it rounds Cape Farewell; melt water from southwestern Greenland joins the West Greenland Current, similarly merging with the Irminger Current (Fig. 1). Melt water from the CAA enters the Labrador Sea through Davis and Hudson straits, either directly or indirectly^49^. The pattern of boundary currents and eddy activity around Greenland and Labrador insures that at least 75 per cent of the freshwater flux from the Greenland ice sheet and CAA eventually winds up in the Labrador Sea (Supplementary Methods). Freshwater and sea ice drained from the Arctic Ocean moves south through Fram Strait and the CAA^37^, also contributing to freshening of the Labrador Sea both remotely and locally^50^,^51^. We estimate that at least 65 per cent of freshwater and sea ice exported from the Arctic Ocean through Fram Strait and the CAA ultimately makes it to the Labrador Sea (Supplementary Methods). Assuming that these estimates are correct, of the 20-mSv freshwater flux increase that we estimate, at least 14 mSv (70%) winds up in the Labrador Sea (Supplementary Methods). Given typical coastal current velocities, most of this freshwater is transported to the Labrador Sea within 3–12 months. Some freshwater from the CAA may take 2–3 years to reach the Labrador Sea due to recirculation and storage in Baffin Bay and/or recirculation in the sub-polar gyre.

### Impact of increased the freshwater flux on deep water formation

**Figure 5:**
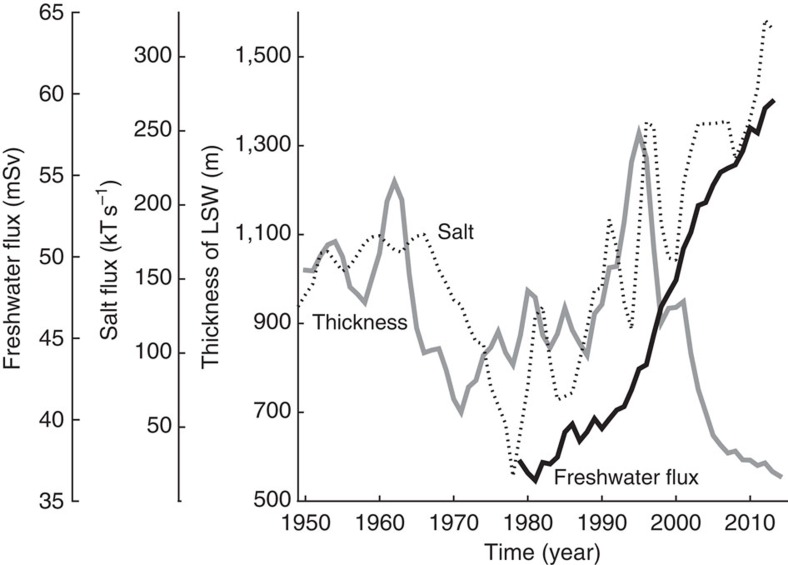
Thickness of LSW and total freshwater flux and salt flux of Irminger Water. Grey solid line indicates the thickness of LSW, black solid line indicates total freshwater flux and dotted line indicates salt flux of Irminger Water. Thickness and salt flux are smoothed with a 3-year running mean. Thickness is obtained from the objective analysis of EN4.0.2 data set from the UK Met Office Hadley Center^52^. Thickness is averaged over 50° N–65° N and 38° W–65° W. Expression of salt flux in terms of freshwater flux is shown in Supplementary Fig. 7.

To investigate effects of increased freshwater flux on deep water formation in the Labrador Sea, we can either look at the mean density of Labrador Sea Water (LSW) within a given depth range or look at the thickness of LSW as defined by a given density range. We used both approaches, obtaining similar results. Figure 5 shows results from the second approach, where we calculate the thickness of LSW, defined by 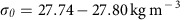, from 1950 to 2013, using the objective analyses of the EN4.0.2 data set from the UK Met Office Hadley Center^52^. The data set includes monthly temperature and salinity, with a spatial resolution of 1° × 1° and 42 depth intervals (5–5,350 m) from 1900 to present. Results for density over a fixed depth range (1,000–2,500 m) are shown in Supplementary Fig. 8.

Figure 5 shows the time series of LSW thickness, compared with our estimate of freshwater flux and with the Irminger salt flux time series. From 1950 to the mid-1990s, Irminger salt flux and LSW thickness are weakly correlated (*R*=0.3, *P*=0.03), and both show multidecadal oscillations, with highs in the 1960s, lows in the 1980s and highs in the 1990s. In particular, LSW thickness increased significantly (by 65%) between 1990 and 1995 when the salt flux increased, consistent with the idea that dense deep water in the Labrador Sea originates from warm, saline North Atlantic water that subsequently experiences winter cooling. However, this relationship begins to break down in the mid- to late-1990s, when the freshwater flux from Greenland and other sources increased rapidly. Since then, LSW thickness decreased continuously, reaching lows not observed since the early 1970s, despite continued high salt flux. One interpretation of this is that the increased freshwater flux has now overwhelmed increased salt flux from the Atlantic, and has begun to influence LSW formation. Recall that the increased salt flux from the Atlantic is accompanied by an increased heat flux (Fig. 3), which promotes melting of marine-terminating outlet glaciers in southern Greenland^29^,^53^ and an increased freshwater flux.

Our data are consistent with recent studies, showing a decline in the thickness of the dense mode of LSW since 1994/95, with a switch to a less dense and presumably fresher and warmer upper mode^54^,^55^. Yashayaev *et al*.^56^ show declining upper salinity since the mid-2000s and suggest that salinity is the controlling factor for ocean stratification in this region. Declining upper layer salinity would weaken or even prevent Labrador Sea convection. However, cold winter air also plays a role in LSW formation. Severe winter conditions and strong air–sea heat exchange for the period 1990–1995 may have contributed to the increased LSW thickness^57^, while milder winter conditions and weaker cooling since 1995 may have contributed to LSW decline^58^. The Labrador Sea is also sensitive to multidecadal climate variations. Hydrographic properties in the Labrador Sea exhibit multidecadal variability that resemble the Atlantic Multidecadal Oscillation and the North Atlantic Oscillation^56^, and these variations are obvious in the flux (Fig. 3) and LSW thickness (Fig. 5) time series. Bidecadal variability in the Labrador Sea forced by volcanic activity has also been proposed^59^. Despite these complications, our data clearly show a steep, recent increase in the freshwater flux into the Labrador Sea and a steep decline in LSW thickness (and density) at the same time (Fig. 5), which is inconsistent with the estimated salt flux into the region. This suggests a potential impact on the formation of North Atlantic Deep Water.

## Discussion

Our reconstructed annual freshwater flux for the sub-polar North Atlantic reaches 60 mSv in 2013, with an increase of 20 mSv in the last two decades (Fig. 4). At least 70 per cent (14 mSv) of this increased freshwater is focused towards the Labrador Sea (Supplementary Methods). This is a minimum estimate since we do not consider other major sources. LSW formation may reflect a delicate balance between this cold freshwater and warm, salty North Atlantic water from the Irminger Current. The flux of freshwater from Greenland may in turn be influenced by warm Atlantic water and its influence on the regional ocean and atmosphere, a potentially important feedback in the system.

Since LSW is an important component of the dense southward return flow of the AMOC, factors influencing LSW formation may in turn have an impact the AMOC. Hosing experiments show different sensitivities of the AMOC to freshwater fluxes at high latitudes^11^,^12^,^13^,^14^,^15^,^16^,^17^. Hu *et al*.^14^ suggest that freshwater inputs much larger than we observe are required. On the other hand, Fichefet *et al*.^11^ suggest that freshwater flux anomalies as small as 15 mSv will affect the AMOC. Brunnabend *et al*.^17^ suggest that freshwater flux anomalies as small as 7 mSv applied over 30 years could have an impact on the AMOC. Different model outcomes partly reflect their spatial resolution, the degree to which freshwater fluxes are focused towards the Labrador Sea, and the timescale over which the anomalous flux is applied. Swingedouw *et al*.^15^ compared different model responses to freshwater release around Greenland, assuming freshwater focusing into the Labrador Sea. They show significant AMOC weakening after several decades with a flux anomaly of 100 mSv.

If our inference that the sub-polar gyre's coastal currents focus melt water from Greenland, CAA glaciers and Arctic sea ice into the Labrador Sea is correct, then present rates of increased freshwater flux may be sufficient to influence convection in the Labrador Sea and, by implication, the AMOC. Northward decreases in heat and salt fluxes across our three sections in southwest Greenland indicate a strong mixing of coastal water and westward advection into the Labrador Sea. Eddy kinetic energy reaches a local maximum offshore Cape Desolation and Paamiut, where a front develops between Irminger Water and fresh shelf water, promoting baroclinic instability and eddy formation; these eddies propagate westwards into the Labrador Sea. Local bathymetric structures, especially the sill at Davis Strait, also promote westward propagation of coastal water from southwestern Greenland. Recent high-resolution eddy-permitting or eddy-resolving numerical models support this type of spatial focusing, and indicate decline or even shutdown of Labrador Sea convection with an enhanced freshwater flux from Greenland^60^ or from the Arctic Ocean through the CAA^61^. Since freshwater lenses can retain their integrity for some time, ‘temporal focusing' may also be important. Summer (June, July and August) freshwater fluxes from Greenland and CAA's ice and snow runoff greatly exceed the annual mean. Summer freshwater flux from Greenland and the CAA increased by ∼50 mSv from mid-late 1990s to 2013, reaching a height of 150 mSv in 2012 (Supplementary Fig. 9), potentially limiting convection during the subsequent winter.

We suggest that recent freshening in the vicinity of Greenland is reducing the formation of dense LSW, potentially weakening the AMOC. Recent observations are beginning to document declines in the AMOC^62^,^63^,^64^, consistent with our hypothesis. Longer time series will be required to confirm this link, but our preliminary results suggest that detailed studies of Labrador Sea hydrography and proximal sources of freshwater, including Greenland, have the potential to improve our understanding of AMOC variability and sensitivity to anthropogenic warming.

## Methods

### GRACE data

The GRACE time series were created via the least squares inversion method described in ref. 24. Release-05 GRACE data from the Center for Space Research were used, with the standard post-processing applied as described in that paper: C_20_ is replaced by Satellite Laser Ranging estimates, a geocentre model is added, GIA is corrected for and the monthly averages of the Atmosphere and Ocean Dealiasing product are restored.

The inversion technique is designed to localize the mass change signal, such that coastal mass loss from Greenland does not leak into the ocean or into interior Greenland because of GRACE's inherently low spatial resolution. Briefly, the method involves breaking Greenland and the surrounding area into pre-defined regions (Greenland drainage basins; Supplementary Fig. 10). Each region is assumed to have a uniform mass distribution when gridded as 1° × 1°-binned kernels. The transformation to degree/order 60 spherical harmonics is then made on each individual regional kernel, resulting in a smoothed version of each region that mimics what GRACE would see from its limited resolution, if a uniform mass of 1 was placed over the kernel, with zeroes elsewhere.

The goal is to find a set of multipliers for each region that most closely describes mass distribution over Greenland, given the assumption of uniform weights across each pre-defined shape. A least squares method is used to fit an optimal multiplier to each basin simultaneously, such that the combination of the multiplier times the smoothed basin kernels best fits the actual (smoothed) GRACE data for that month. An optimal amount of process noise is added to stabilize the solution^24^.

The GRACE mass balance in this paper is the sum of the individual signals from the 16 Greenland regions (Supplementary Fig. 10).

### Irminger Water heat and salt flux analysis

Details of the data collection and analysis are discussed in Myers *et al*.^27^ and summarized here. Before 1984, the estimates are based on a climatological analysis of the Labrador Sea. The 1984–2013 observations are collected on a set of standard sections by the Danish Meteorological Institute. Each section (Fig. 1) involves the same five stations; however, in some years only three or four stations could be occupied. The sections are occupied annually in most years, in late June or early July. Direct sampling using bottles was performed in 1984–1987, while conductivity–temperature–depth data were collected in later years. We carry out our analysis on the upper 700 m, the deepest depth common to all years, binned on a 2-m vertical grid. For current motions, we determine the geostrophic velocity, relative to 700 dbar (∼700 m depth) or the bottom in shallower water, for each pair of stations at each depth, and add an estimate of the barotropic velocity^33^. If data are missing, we do not include that point in the calculation. We calculate heat flux (*Q*_t_) and salt flux (*Q*_s_) at each depth and then sum those whose temperature and salinity are consistent with Irminger Water to obtain the total transport:


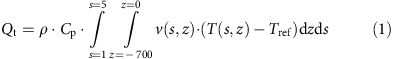



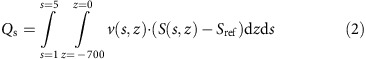


where *ρ* and *C*_p_ are ocean water density and heat capacity, respectively; *v*(*s*,*z*), *T*(*s*,*z*) and *S*(*s*,*z*) are velocity, temperature and salinity in station *s* at depth *z,* respectively; *T*_ref_ is the reference temperature (0 °C) and *S*_ref_ is the reference salinity (34.80). Here we choose a broad definition including both pure and modified Irminger Water, with temperatures warmer than 3.5 °C and salinity higher than 34.88 (ref. 27).

### Freshwater flux

To estimate the freshwater flux from Greenland, we first use a simple constant acceleration model to fit the monthly GRACE mass balance data:


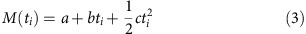


where *M*(*t*_*i*_) (*i*=1,2,3…..*n*) are GRACE monthly solutions, *a* is the initial mass of Greenland, *b* is the initial mass balance and *c* is the acceleration term. Given the estimated parameters, the mass balance (MB) of Greenland can be represented by:


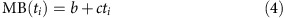


The start time of recent accelerated mass loss is the time *t*_*i*_ when MB(*t*_*i*_) is zero. The mass balance of Greenland is:


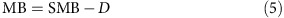


where SMB is surface mass balance and *D* is discharge, related to freshwater flux (FWF) by:


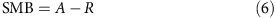



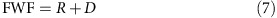


where *A* is the accumulation and *R* is runoff.

We then calculate freshwater flux from Greenland using the above relations, rewriting them as:


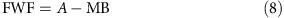


where accumulation (*A*) is predicted by RACMO2.3 and MB is estimated from the GRACE data. Note that accumulation is defined over ice and tundra, and mass balance is the total mass balance of Greenland, including ice and tundra. Therefore, freshwater flux from Greenland is composed of ice mass loss and tundra runoff (Supplementary Fig. 11). Mass balance is considered equal to zero before the recent acceleration phase, beginning in 1996. Since mass balance is the long-term average, accumulation is smoothed with 5-year running average.

For the CAA, we assume FWF=*R* when estimating freshwater flux since ice discharge from the CAA is negligible compared with runoff^44^. Thus, freshwater flux from the CAA is derived from runoff predicted by RACMO2.3. Note that both ice runoff and tundra runoff are considered in the freshwater flux calculation (Supplementary Fig. 12).

For Arctic sea ice, we focus just on recent accelerated melting of multi-year ice, which results in the loss of ice area and extent, rather than the much larger contribution from the annual freeze–thaw cycle, which forms significant freshwater through fractionation (Supplementary Methods), but is more difficult to quantify with remote methods. We use three data sets (area, extent and volume; see Supplementary Methods and Supplementary Fig. 5) to estimate freshwater flux from accelerated melting of Arctic sea ice. All three approaches give similar results (Supplementary Fig. 6). The one based on volume is shown in Fig. 4. To convert area and extent to mass, we assume that sea ice thickness is 2 m (ref. 47) and sea ice density is 900 kg m^−3^. Annual melting of Arctic sea ice is estimated by fitting annual minimum Arctic sea ice mass estimates with a linear state space model (Supplementary Methods).

## Additional information

**How to cite this article:** Yang, Q. *et al*. Recent increases in Arctic freshwater flux affects Labrador Sea convection and Atlantic overturning circulation. *Nat. Commun.* 7:10525 doi: 10.1038/ncomms10525 (2016).

## Supplementary Material

Supplementary Information Supplementary Figures 1-12, Supplementary Note 1, Supplementary Methods and Supplementary References.
