# Corrigendum: Recent increases in Arctic freshwater flux affects Labrador Sea convection and Atlantic overturning circulation

**DOI:** 10.1038/ncomms13545

**Published:** 2016-11-17

**Authors:** Qian Yang, Timothy H. Dixon, Paul G. Myers, Jennifer Bonin, Don Chambers, M. R. van den Broeke, Mads H. Ribergaard, John Mortensen

Nature Communications 7: Article number: 10525; DOI: 10.1038/ncomms10525 (2016); Published: 01 22 2016; Updated: 11 17 2016

The authors wish to add ‘Mads H. Ribergaard' and ‘John Mortensen' to the author list of this Article for the provision of oceanographic data. This has now been updated in both the PDF and HTML versions of the Article.

