# Supplementary material for: Recent increases in Arctic freshwater flux affects Labrador Sea convection and Atlantic overturning circulation
Source: Nat Commun. 2016 Jan 22;7:10525. doi: 10.1038/ncomms10525 (PMC4736158; doi:10.1038/ncomms10525)
Supplement: Supplementary Information — Supplementary Figures 1-12, Supplementary Note 1, Supplementary Methods and Supplementary References. [file ncomms10525-s1.pdf]

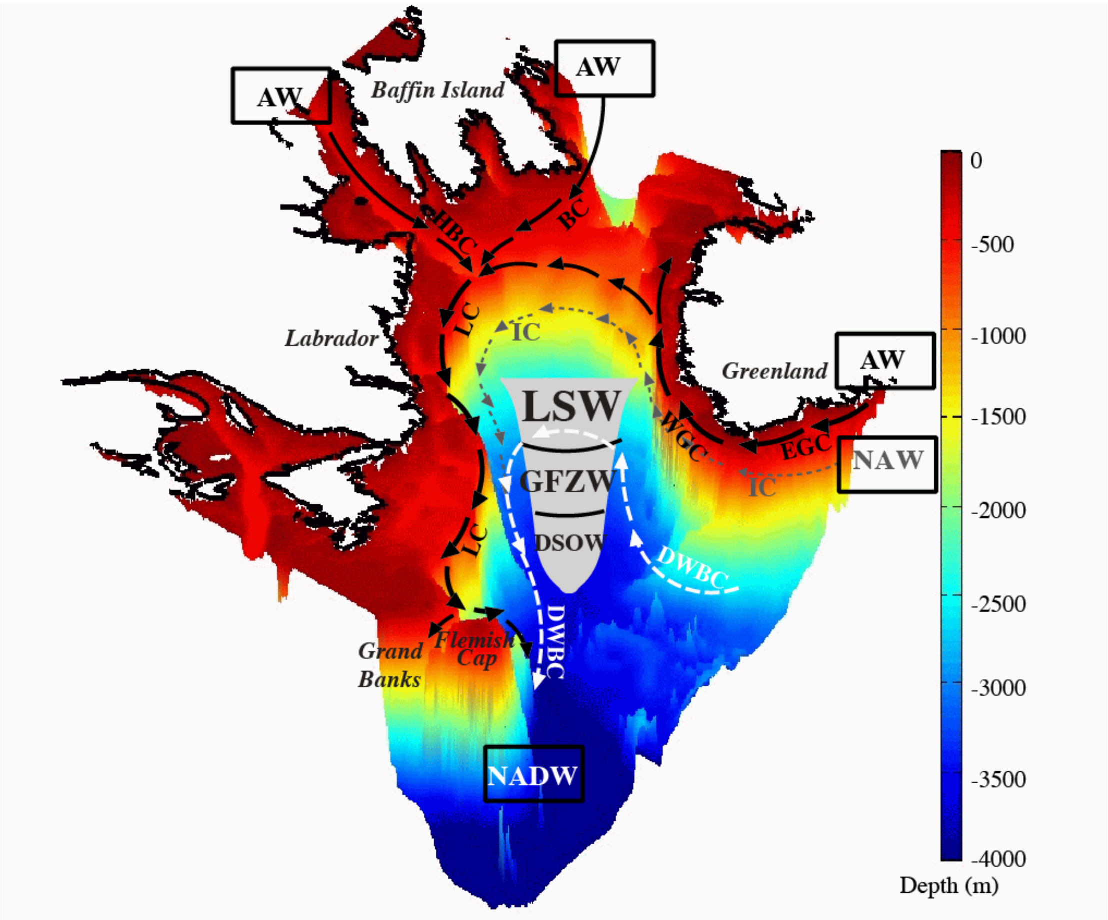

Supplementary Figure 1. Simplified sketch illustrating 3-D structure of the Labrador Sea and major currents and water masses. Black boxes represent water masses input to and output from the Labrador Sea along specific currents. Arrows represent ocean currents at different depths: black solid arrows represent surface ocean currents carrying cold and fresh Arctic Water; grey dashed arrows represent subsurface ocean current carrying warm and salty North Atlantic Water; white dashed arrows represent the Deep Western Boundary Current that moves North Atlantic Deep Water southward. EGC is East Greenland Current, WGC is West Greenland Current, HBC is Hudson's Bay Current, BC is Baffin Current, LC is Labrador Current, IC is Irminger Current, DWBC is Deep Western Boundary Current. AW is Arctic Water, NAW is North Atlantic Water, LSW is Labrador Sea Water, GFZW is Gibbs Fracture Zone Water, DSOW is Denmark Strait Overflow Water, NADW is North Atlantic Deep Water. Not shown are the geographic sources of Arctic Water, which include the Greenland ice sheet, Arctic Ocean, Canadian Arctic Archipelago, and Hudson Bay, or the types of ice and water masses that contribute, which include glaciers and ice sheets, rivers, sea ice, and precipitation. Also not shown are the major time scales for significant variability, which include annual (summer ice melting, winter cooling

and convection) and decadal to multi-decadal (e.g., North Atlantic Oscillation, Atlantic Multi-decadal Oscillation).

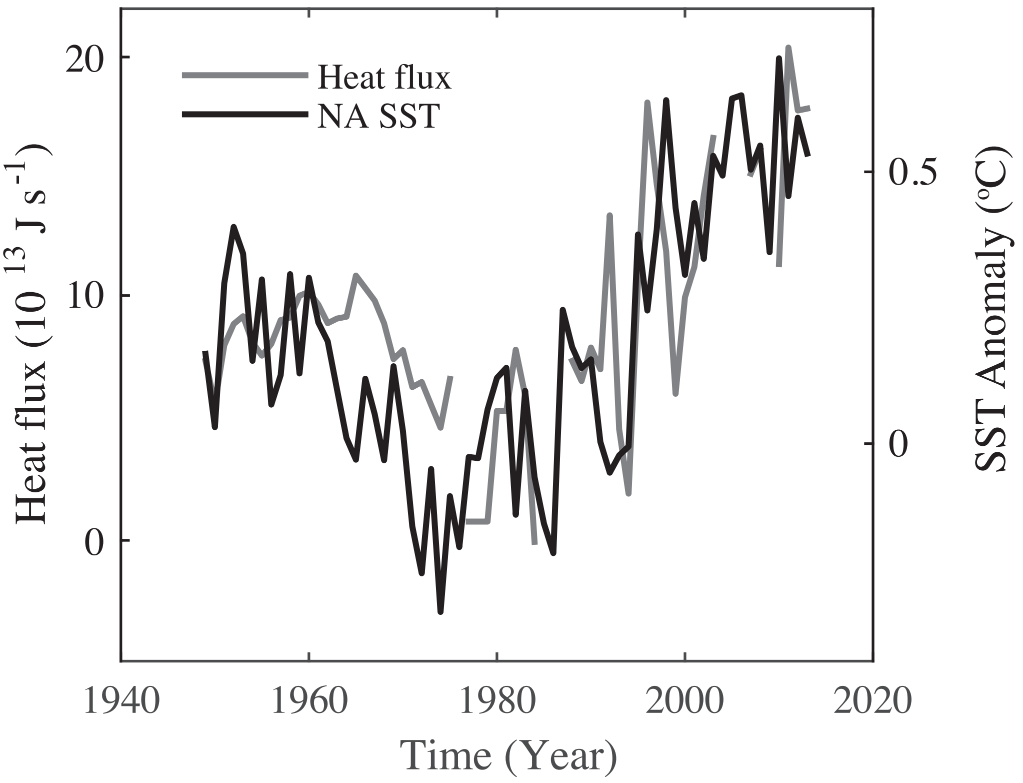

Supplementary Figure 2. Comparison of Irminger Water heat flux and North Atlantic SST anomaly over the period 1949 – 2013. Grey line indicates Irminger Water heat flux, black line indicates North Atlantic SST anomaly. We use the HADISST dataset to compute SST anomaly. We compute the annual average SST anomaly over a broad area of the North Atlantic, using as boundaries 0° – 60° North latitude and 0° – 80° West longitude, relative to the average temperature for the period 1901 to 1970. Average North Atlantic SST anomaly shows a strong ($R = 0.68$) and significant ($P = 0.001$) correlation with our Irminger heat flux data for the 1949 to 2013 period, with both indices increasing strongly after 1995.

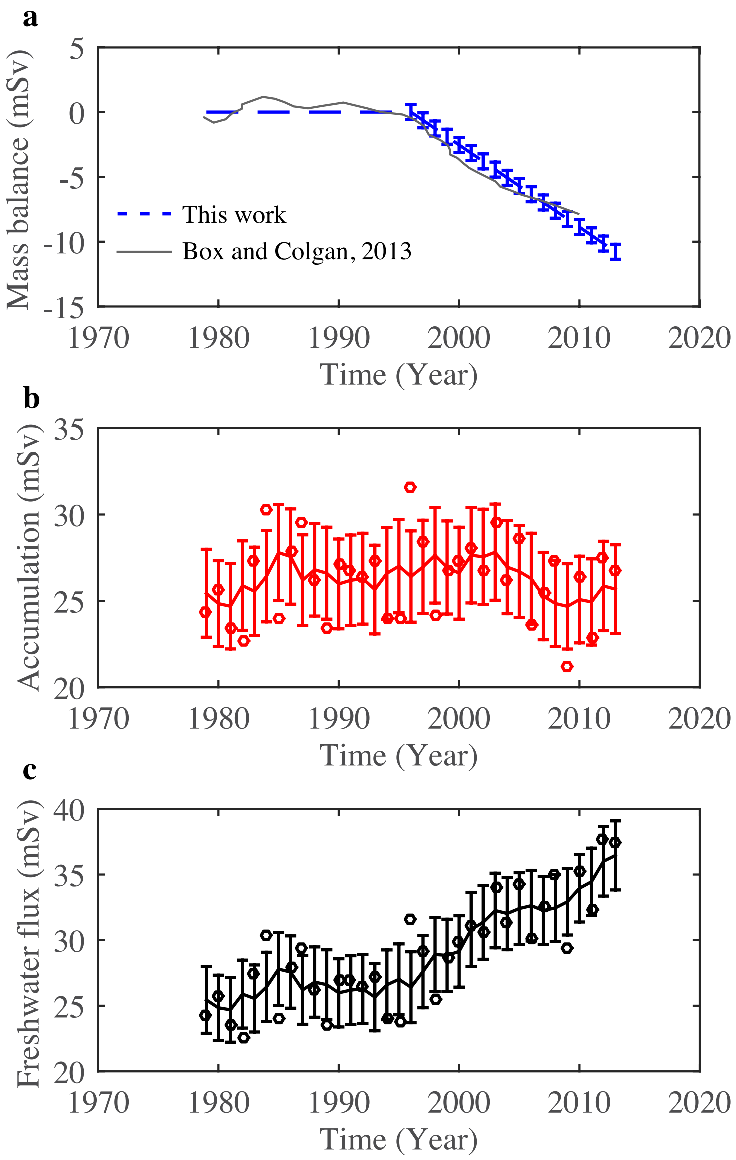

Supplementary Figure 3. Freshwater flux from Greenland estimated from mass balance and accumulation. (**a**) Greenland mass balance**.** Estimate from this study (blue dashed line) is compared with estimate from Box and Colgan[1]. Blue error bars indicate uncertainty of mass balance, estimated at 95% confidence level. (**b**) Greenland accumulation. Red circles represent annual value. Red solid line represents 5-year running average. Red error bars indicate uncertainty of smoothed accumulation, which is approximated by uncertainty of accumulation modeled by RACMO2.3 (±9%). (**c**) Freshwater flux from Greenland. Black circles represent freshwater flux from mass balance and annual accumulation (see equation 1). Black solid line represents freshwater flux from mass balance and smoothed accumulation. Black error bars indicate propagated uncertainty.

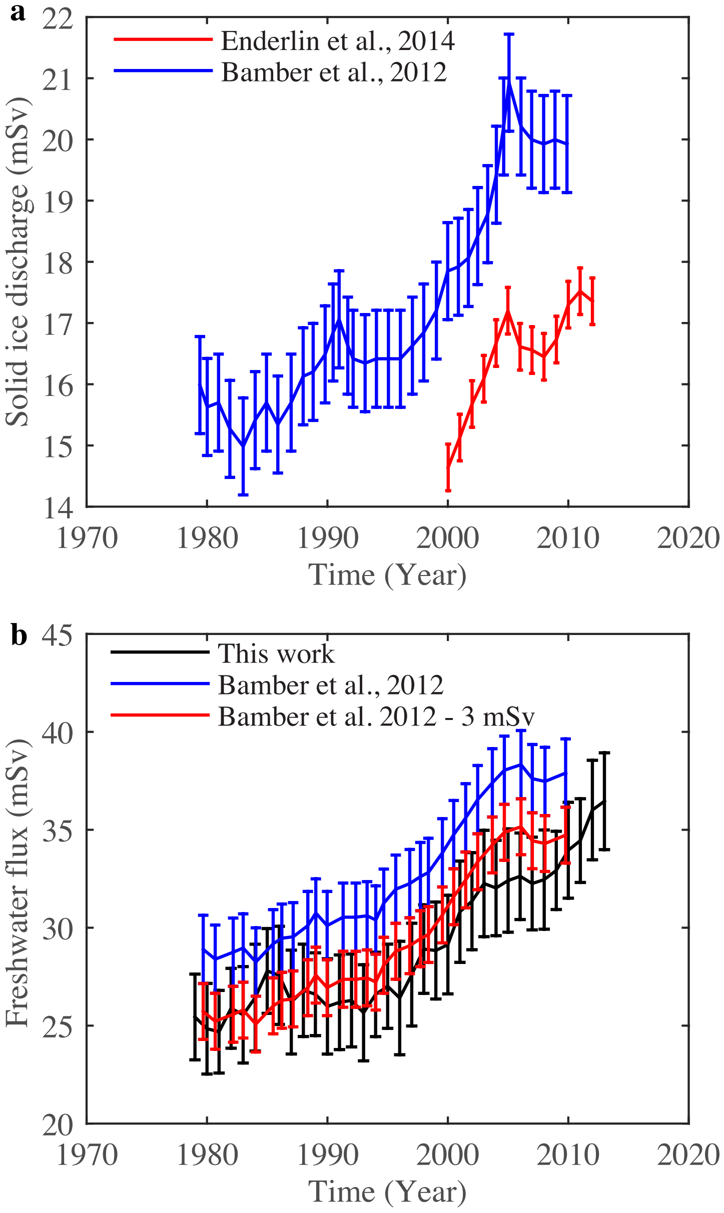

Supplementary Figure 4. Comparison between estimates of freshwater flux from Greenland. (**a**) Comparison between two estimates of solid ice discharge from Greenland. Blue line from Bamber *et al.*[2], red line from Enderlin *et al.*[3]. The estimate from Bamber *et al.*[2] required extrapolation to cover all Greenland discharge and is ~3 mSv (~100 km ^{3} yr $^{-1}$) larger than the more recent estimate from Enderlin *et al.*[3]. (**b**) Comparison of three estimates of freshwater flux from Greenland: black line (this study), blue line (Bamber *et al.*[2]), red line (Bamber *et al.*[2]) with correction following Enderlin *et al.*[3].

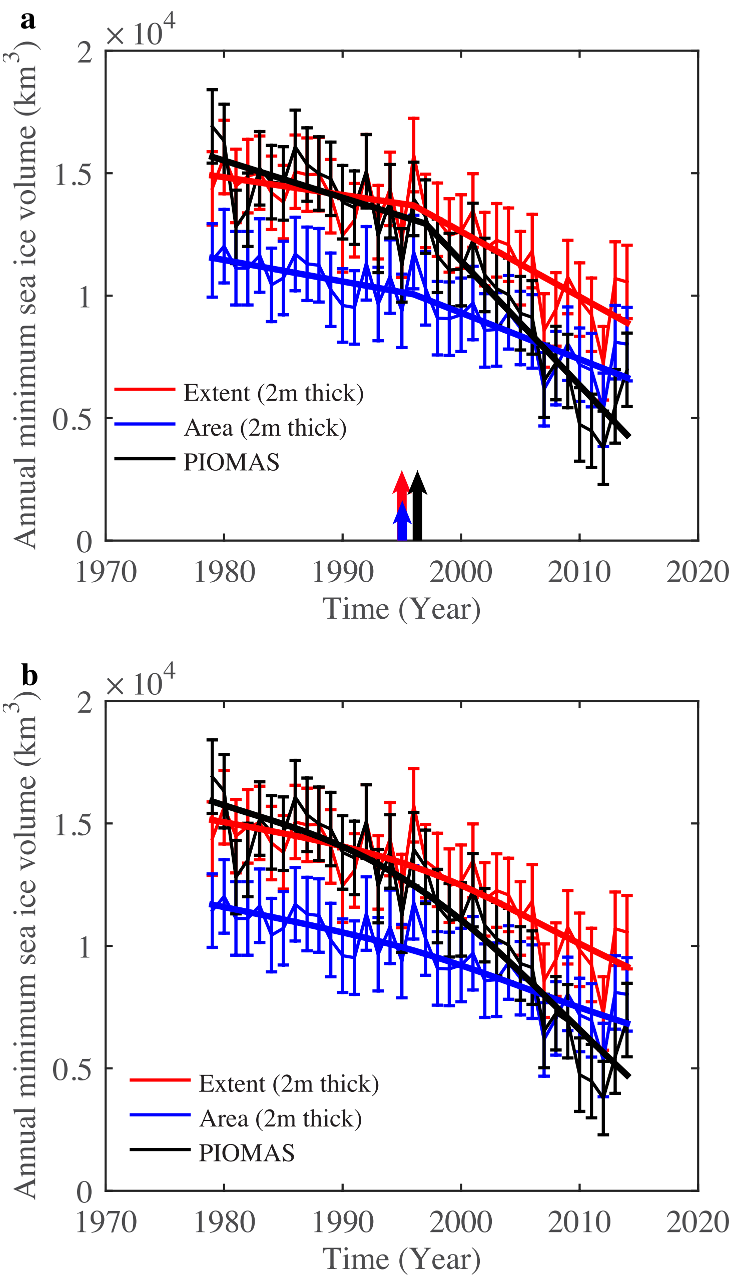

Supplementary Figure 5. Three estimates of the annual minimum Arctic sea ice volume time series. Red line represents estimate based on ice extent assuming 2 m thickness. Blue line represents estimate based on ice area assuming 2 m thickness. Black line represents volume modeled by the Pan-Arctic Ice Ocean Modeling and Assimilation System (PIOMAS)[4]. Error bars represent the uncertainty of annual minimum Arctic sea ice volume (1500 km^3). (**a**) Three time series described above are fit with a two-slope model (thick solid line) (Supplementary methods). Arrow marks the onset time of accelerated melting derived from three data sets: 1996 for ice extent and ice area data sets, 1997 for PIOMAS data set. (**b**) Three time series are fit with the linear state space model (thick solid line) (Supplementary methods).

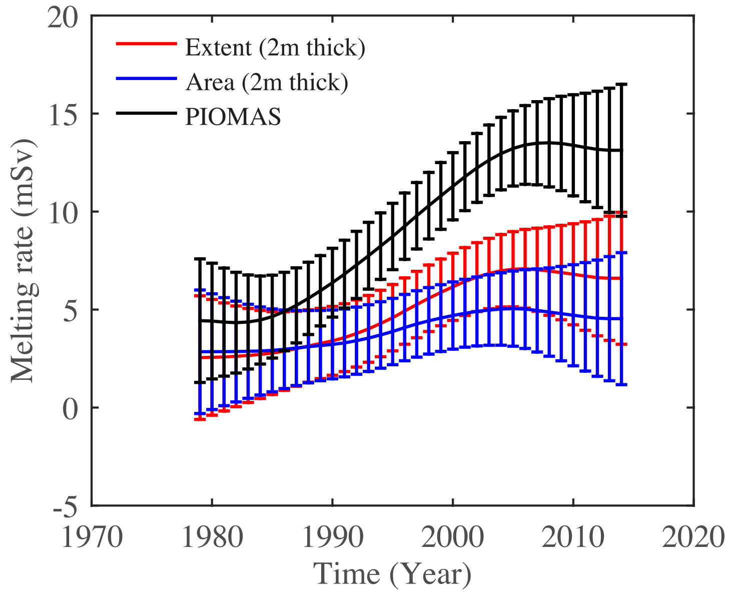

Supplementary Figure 6. Long term melting rate of Arctic sea ice from three data sets. Red line represents estimate based on ice extent data set. Blue line represents estimate based on ice area data set. Black line represents estimate from the Pan-Arctic Ice Ocean Modeling and Assimilation System (PIOMAS) data set. The melting rate is estimated using the linear state space model (Supplementary Fig. 5b). Error bars indicate uncertainty at 95% confidence level.

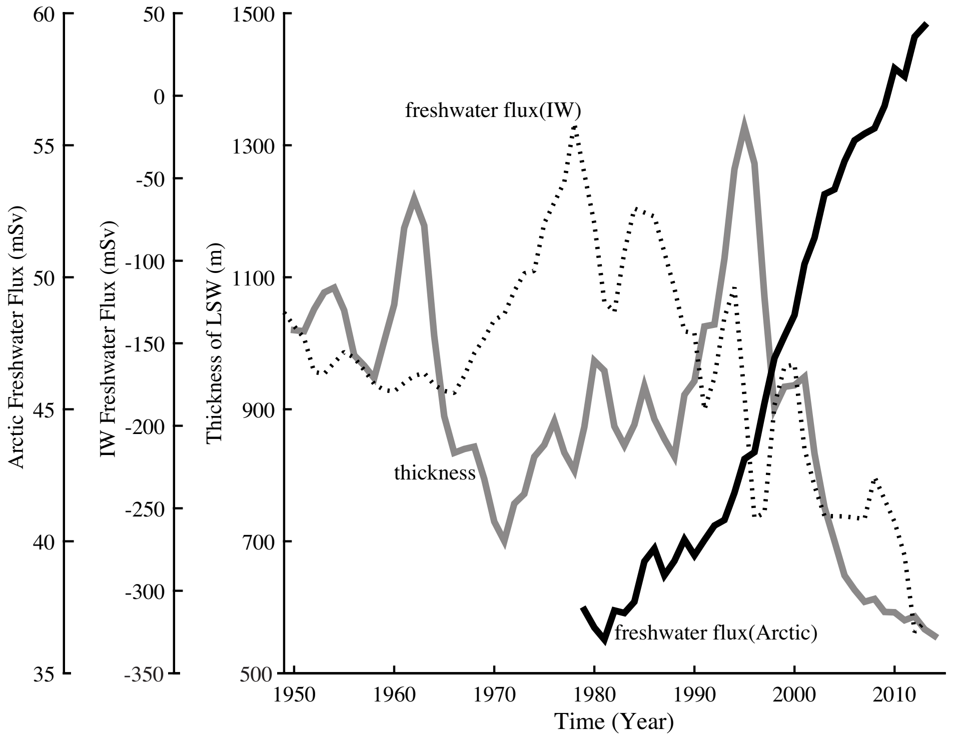

Supplementary Figure 7. Similar to Fig. 5 except salt flux of Irminger Water is expressed in terms of freshwater flux. Grey solid line represents thickness of Labrador Sea Water. Black solid line represents Arctic freshwater flux (the sum of freshwater flux from Greenland, the Canadian Arctic Archipelago and Arctic sea ice). Dotted line represents freshwater flux of Irminger Water (IW). Salt flux is converted to freshwater flux using 34.80 as a reference salinity.

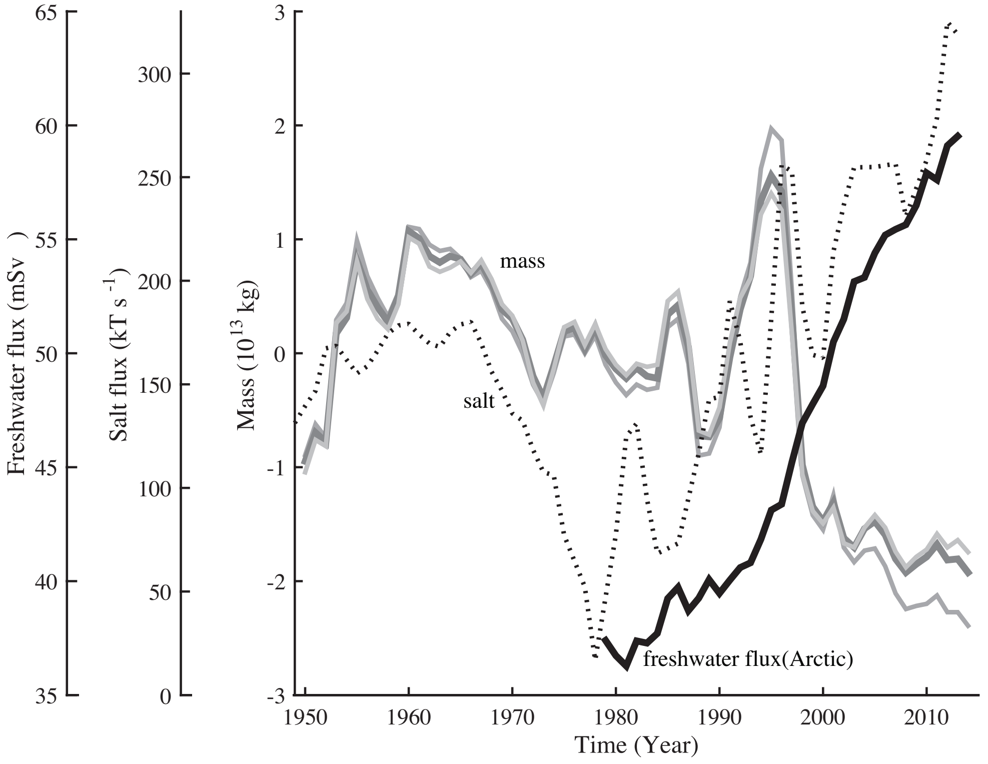

Supplementary Figure 8. Similar to Fig. 5 except grey solid line represents the mass of Labrador Sea Water. Black solid line represents Arctic freshwater flux (the sum of freshwater flux from Greenland, the Canadian Arctic Archipelago and Arctic sea ice). Density of Labrador Sea Water is obtained from the objective analyses of EN4.0.2 dataset from the UK Met Office Hadley Center[5]. Mass values are calculated by integrating density with volume between 50° N – 65° N, 38° W – 65° W and three depth range (900 – 2400 m, 1000 - 2500 m and 1100 – 2600 m), relative to the mean of 1950 – 2006.

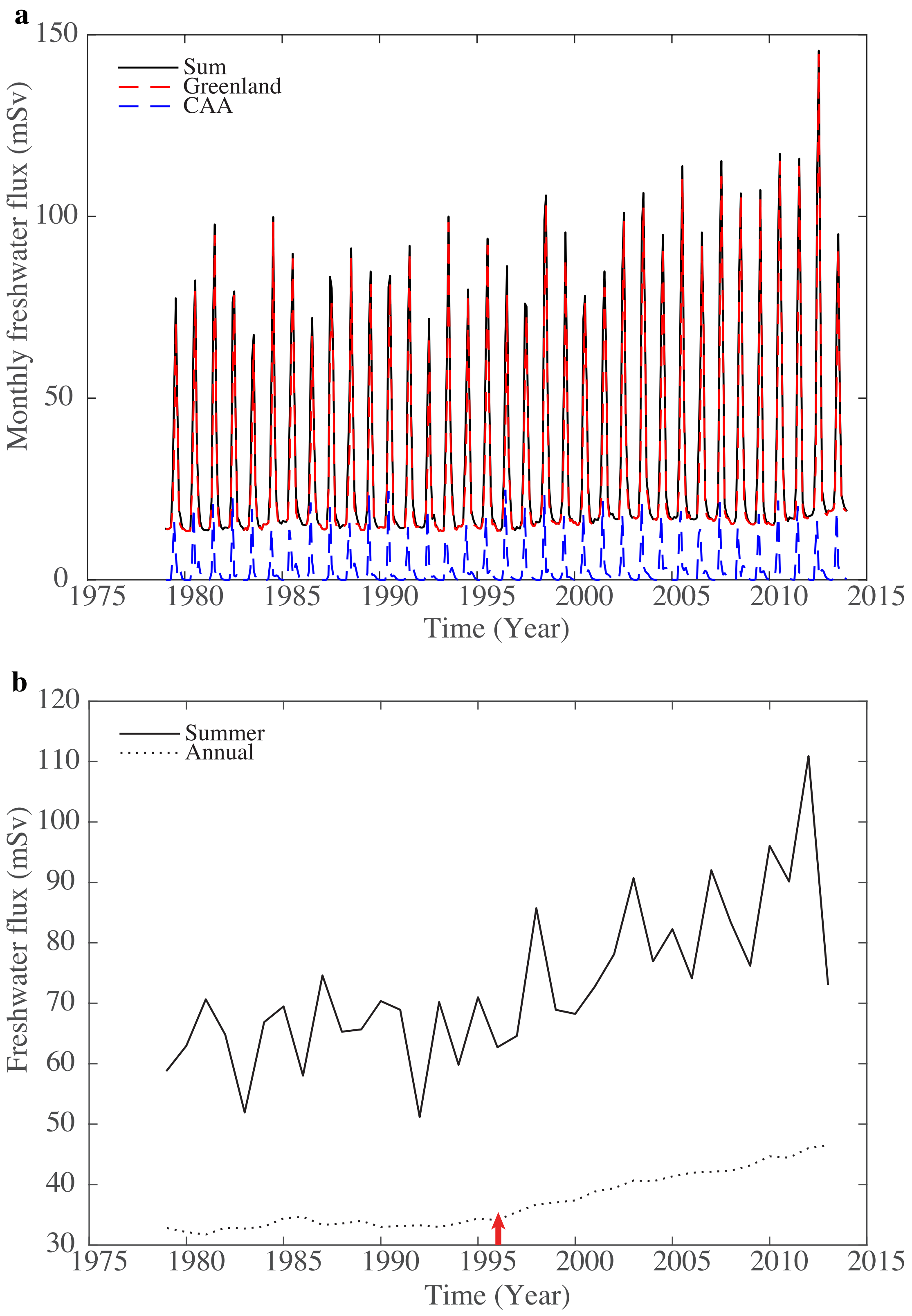

Supplementary Figure 9. Seasonal variation of freshwater flux from Greenland and the Canadian Arctic Archipelago. (**a**) Monthly freshwater flux from Greenland (GL), Canadian Arctic Archipelago (CAA) and their total for 1979 – 2013. Freshwater flux from Greenland and CAA peaks in July. Freshwater flux since 2002

has exceeded 100 mSv for about a month a year 9 times, with 2012 having the highest value (150 mSv). (**b**) Total summer (June, July and August) freshwater flux (solid line) compared to long-term averaged annual freshwater flux (dashed line). Note that summer freshwater flux increased significantly about the time when GRACE data and a simple model of constant acceleration suggest that the current phase of accelerated Greenland mass loss began (red arrow) (see Fig. 2).

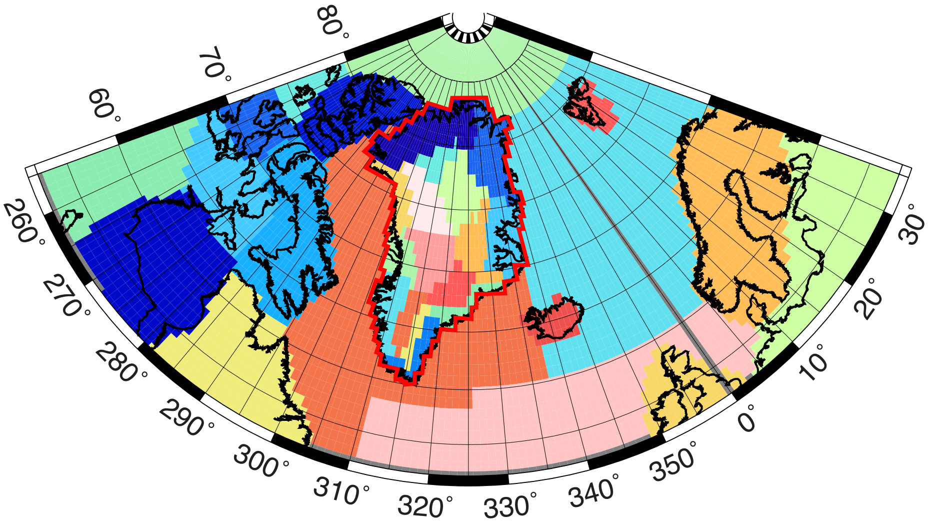

Supplementary Figure 10. Predefined regions used in this paper to localize the GRACE mass change signal. Greenland is outlined with solid red line.

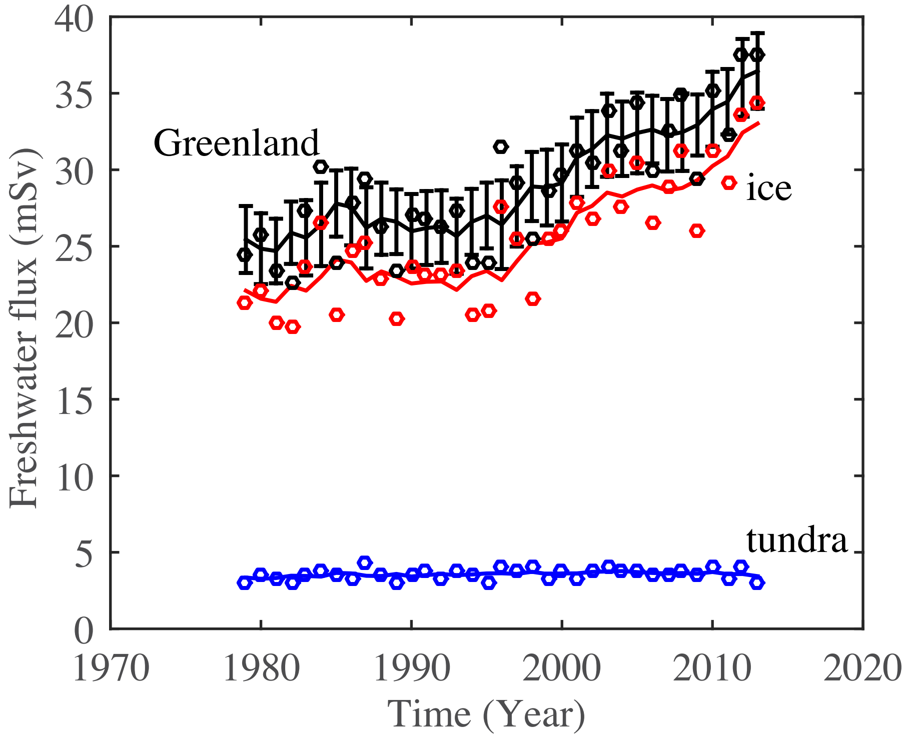

Supplementary Figure 11. Freshwater flux from Greenland and its two components. Black circles and solid line represent freshwater flux from Greenland. Red circles and solid line represent freshwater flux component from ice mass loss. Blue circles and solid line represent freshwater flux component from tundra runoff. Circles represent annual value and solid line represents 5-year running average. Black error bars indicate propagated uncertainty (Supplementary Fig. 3).

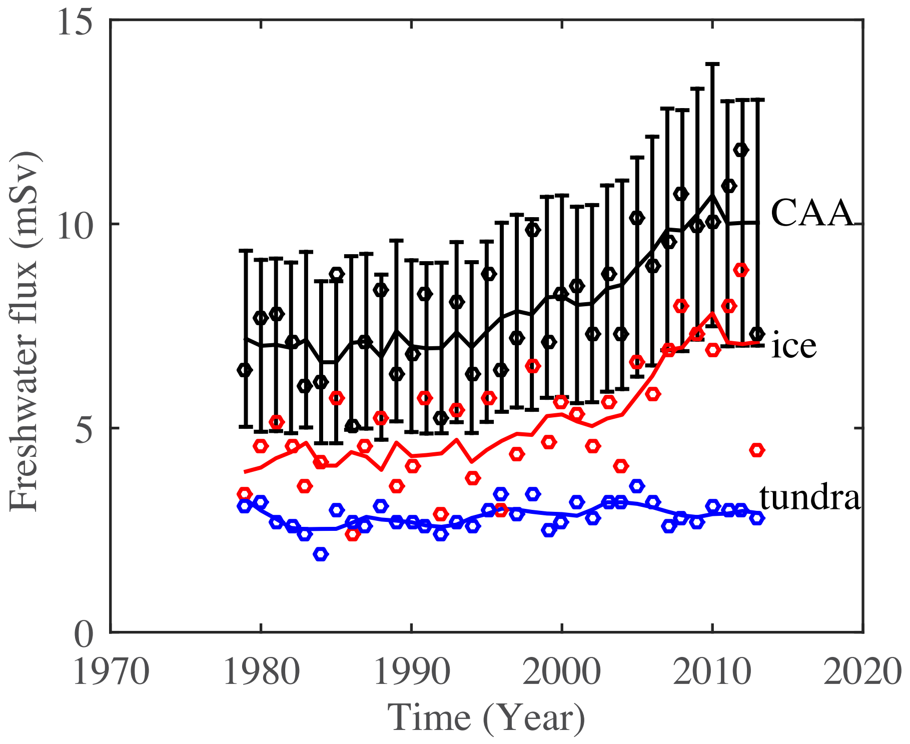

Supplementary Figure 12. Freshwater flux from the Canadian Arctic Archipelago and its two components. Black circles and solid line represent freshwater flux from the Canadian Arctic Archipelago (CAA). Red circles and solid line represent freshwater flux component from ice mass loss. Blue circles and solid line represent freshwater flux component from tundra runoff. Circle represents annual value and solid line represents 5-year running average. Black error bars indicate uncertainty of smoothed freshwater flux from CAA, approximated by uncertainty of runoff modeled by RACMO2.3, believed to be accurate to ±30%[6].

Supplementary Note 1: Additional information on Fig. 4

Figure 4 shows the sum of freshwater flux from Greenland, the Canadian Arctic Archipelago and Arctic sea ice. Grey shading in Fig. 4 indicates propagated uncertainty. It is computed by taking the quadratic sum of the uncertainty associated with each freshwater flux estimate (Supplementary Figs 3, 6 and 12), and then taking the square root of the sum.

Supplementary Methods

Freshwater flux from Greenland

Freshwater flux from Greenland (FWF_{GL}) is described by Bamber *et al.*[2] :

$$FWF_{GL} = A_{GL} - MB_{GL} \quad (1)$$

where $A_{GL} = A_{ice} + A_{tundra}$ and $MB_{GL} = MB_{ice} + MB_{tundra}$

A_{GL} is the total accumulation in Greenland, A_{ice} is accumulation on ice and A_{tundra} is accumulation on tundra. MB_{GL} is the total mass balance of Greenland, MB_{ice} is the ice mass balance and MB_{tundra} is the snow mass balance on tundra. Since A_{GL} can be estimated from RACMO2.3 (precipitation minus sublimation/evaporation) and MB_{GL} can be estimated from GRACE observations, we can estimate the freshwater flux from Greenland directly with equation (1). Note that the accumulation predicted by RACMO2.3 is variable from year to year. We therefore smooth the accumulation with a 5-year running average (both values are shown in Supplementary Fig. 3).

We then examined two components of freshwater flux from Greenland (FWF_{GL}), namely freshwater flux from ice mass loss (FWF_{ice}) and freshwater flux from snow melt on tundra (FWF_{tundra}) (Supplementary Fig. 11):

$$FWF_{ice} = R_{ice} + D_{ice} \quad (2)$$

$$FWF_{tundra} = R_{tundra} \quad (3)$$

where R_{ice} is ice runoff, D_{ice} is ice discharge and R_{tundra} is tundra runoff.

FWF_{GL} is already estimated using equation (1) and R_{tundra} is given by RACMO2.3 directly. Thus, we can estimate FWF_{ice} by subtracting FWF_{tundra} from FWF_{GL}.

Freshwater flux from the Canadian Arctic Archipelago (CAA)

Like FWF_{GL}, freshwater flux from the CAA (FWF_{CAA}) is composed of freshwater flux from ice mass loss (FWF_{ice}) and freshwater flux from snowmelt on tundra (FWF_{tundra}) (Supplementary Fig. 12). Glaciers in the CAA are mainly land-terminating, so freshwater flux by ice discharge is small (5 ± 2 Gt yr^{-1}/ 0.16 ± 0.06 mSv)[7]. Thus, we only consider ice runoff (R_{ice}) and neglect ice discharge (D_{ice}) for the FWF_{ice} calculation (equation 2). FWF_{tundra} thus equals tundra runoff (R_{tundra}) (equation 3). FWF_{CAA} is then derived from runoff predicted by RACMO2.3.

Changes in freshwater flux from Arctic sea ice

Freshwater sources in the Arctic Ocean include runoff from rivers and streams, ground water discharge, the difference between precipitation and evaporation ($P - E$) and sea ice formation, which forms fresh water through fractionation. All of these sources are thought to be freshening the Arctic Ocean[8].

Freshwater is exported from the Arctic Ocean as liquid water and sea ice, mainly through Fram Strait, Nares Strait and the CAA. Freshwater fluxes from the Arctic Ocean far exceed fluxes from Greenland or melting of CAA glaciers, but are also difficult to quantify. Annual fluxes through Fram Strait are thought to be about ~2800 km^3 and ~1900 km^3 of liquid freshwater and sea ice respectively (~140 mSv total freshwater exported to the Nordic Seas and Labrador Sea) while annual fluxes through the CAA (and subsequently Davis Strait) are ~2900 km^3 and ~320 km^3 of liquid freshwater and sea ice respectively (~100 mSv total freshwater)[8]. These recent estimates do not show significant change over the last few decades, but the uncertainties are quite large, of the order of the changes we observe for Greenland (Fig. 4).

Arctic sea ice contributes to freshwater flux in several ways. It is useful to consider two components. The first component is associated with the annual freeze-thaw cycle that fractionates sea water into freshwater and brine (since the freezing point of brine is lower than freshwater; see Aagaard and Carmack[9] for a review). The solid ice remains at the surface, while the liquid brine sinks, some of which is subsequently exported from the Arctic to form a component of deep water. Most of the ice melts the following summer, contributing significant freshwater. However, some ice may remain unmelted, forming multi-year ice. A large reservoir of thick multi-year ice may eventually form. If the system is in steady state, it is mainly new ice that melts each summer and contributes to freshwater flux.

The second component represents additional ice that melts during periods of extended multi-year warming. If previously accumulated multi-year ice begins to melt, sea ice volume decreases year by year. Here, we ignore the first (larger) component, because it is difficult to calculate, and focus just on changes in freshwater flux due to accelerated melting and export of sea ice.

Another variable to consider is the partitioning between freshwater that is exported from the Arctic Ocean, and freshwater that is retained. The CCSM4 climate model suggests that increased import of freshwater into the Arctic Ocean and increased sea ice melting forces increased export of freshwater[10]. However, decadal freshening of the Arctic has been observed since 2000, indicating that some of the increased fresh water must also be retained, at least temporarily, possibly influenced by decadal changes in wind stress[8, 11]. Additional studies are required to refine our picture of freshwater sinks and sources.

We use the annual minimum of Arctic sea ice volume, and its long term change, to estimate changes in the freshwater flux from Arctic sea ice. Three data sets (sea ice volume, extent and area) are used. We obtained the monthly Arctic sea ice volume time series from the Pan-Arctic Ice Ocean Modeling and Assimilation System (PIOMAS)[4]. Monthly Arctic sea ice extent and sea ice area time series are obtained from the National Snow and Ice Data Center (NSIDC)[12]. To convert extent and area to volume we assume the average thickness of Arctic sea ice is 2 m.

Many studies report a long-term decline in Arctic sea ice[13-15]. The sea ice data compiled here also show a clear trend of accelerating loss, with the loss rate increasing in the 1990s (Supplementary Fig. 5). To determine the timing of this change more accurately, we fit all three time series with a two-slope model, where the trend change occurs at a ramp time. We conducted a one dimensional grid search from 1979 to 2013 with 1 year spacing to determine the best-fit ramp time. Our results suggest that the melting rate of Artic sea ice started to increase around 1996 (based on the ice extent and area data sets) or 1997 (based on the ice volume data set) (Supplementary Fig. 5a), in agreement with Comiso *et al.*[16].

The two-slope model is good at detecting the onset time of accelerated melting, but poorly describes the time-varying melt rate. To better estimate this rate, we also fit the three time series with a linear state space model, described below.

A general linear state space model can be represented by an observation equation and a state evolution equation as[17]:

$$\mathbf{y}_t = \mathbf{F}_t \mathbf{x}_t + \mathbf{v}_t \quad (4)$$

$$\mathbf{x}_t = \mathbf{G}_t \mathbf{x}_{t-1} + \mathbf{w}_t \quad (5)$$

where $\mathbf{y}_t$ is the observation vector at time t (t = 1,2,3,...n), $\mathbf{x}_t$ is the state vector, $\mathbf{F}_t$ is the measurement matrix and $\mathbf{G}_t$ is the state transition matrix for the time step from time t to time $t + 1$. $\mathbf{v}_t$ and $\mathbf{w}_t$ are assumed to be Gaussian with zero mean and measurement noise covariance matrix $\mathbf{V}_t$ and process noise covariance matrix $\mathbf{W}_t$.

In our analysis, $\mathbf{y}_t$ is a 1 × 1 matrix and equals annual minimum Arctic sea ice volume. $\mathbf{x}_t = \begin{bmatrix} \mu_t & \alpha_t \end{bmatrix}^{\mathrm{T}}$, where μ_t is the initial volume state, α_t is the melting rate state. $\mathbf{V}_t$ is a 1 × 1 matrix and equals the observation uncertainty (1500 km ^{3}). We use the same strategy described in Laine *et al.*[18], defining **F**, **G** and **W** to be time-invariant, so they can be represented by:

$$\mathbf{F} = \begin{bmatrix} 1 & 0 \end{bmatrix} \quad (6)$$

$$\mathbf{G} = \begin{bmatrix} 1 & 1 \\ 0 & 1 \end{bmatrix} \quad (7)$$

$$\mathrm{diag}(\mathbf{W}) = \begin{bmatrix} 0 & \sigma_{\mathrm{rate}}^2 \end{bmatrix} \quad (8)$$

σ_{rate} describes allowed change of sea ice volume in a year, with units of km ^{3} yr $^{-1}$. Here, we assume σ_{rate} = 40 km ^{3} yr $^{-1}$. This value balances the trade-off between goodness of fit and smoothing. We then adopt the Kalman filtering technique to estimate the time-dependent state vectors described in the above state space model. We use the software described in Laine *et al.*[18] to implement the Kalman filter.

Supplementary Fig. 5b shows the annual minimum sea ice volume time series and the linear state space model for the three data sets. Supplementary Fig. 6 shows the estimated long-term freshwater flux (mSv) from Arctic sea ice for the three data sets. Note that the freshwater flux from Arctic sea ice is calculated by multiplying the estimated melting rate (km ^{3} yr $^{-1}$) and the density of sea ice (900 kg m $^{-3}$). Melting rate derived from the volume data set is somewhat higher compared to the other two data sets. However, all three data sets show accelerated melting beginning between 1990 and 2000.

Portion of increased freshwater flux that reaches the Labrador Sea

We estimate ~20 mSv of increased freshwater flux into the sub-polar North Atlantic over the last two decades, focusing on three sources that are likely to influence Labrador Sea convection and can be estimated by remote techniques: the Greenland Ice Sheet (GrIS), glaciers in the CAA and changes in Arctic sea ice. We recognize that there are additional freshwater sources such as river runoff and $P - E$, and that these may also have increased in the last few decades[19] but are difficult to quantify[20]. Also, our sea ice change estimate does not capture all the freshwater flux associated with sea ice formation. Hence our estimate of changes in freshwater flux is a minimum estimate. However, it is also important to determine what fraction of our freshwater flux change estimate winds up in the Labrador Sea.

On the east side of Greenland, Arctic Ocean freshwater (liquid freshwater plus sea ice) is exported through Fram Strait. The sea ice melts in the East Greenland Current (EGC), adding to the liquid component. Some of the Arctic freshwater is lost to the Nordic Seas, but the amounts are not well known. Limited *in situ* data may not capture annual or longer term variation, but do allow a crude estimate of the partitioning of freshwater flux during the sampling period through several key flux gates. Using a reference salinity of 35.20, Dickson *et al.*[21] estimate that 148 mSv of freshwater (liquid freshwater plus sea ice) is exported from Fram Strait. This represents freshwater from all sources, including river runoff and $P - E$, but in the calculations below we assume that freshwater sourced just from Arctic sea ice is similarly partitioned. Note that the numbers change if a different reference salinity is used, however overall partitioning is less affected. 51 mSv of the freshwater is exported directly to the deep Atlantic in dense water overflows, leaving 97 mSv in the EGC, or about 65% of the original flux through Fram Strait. An additional 54 mSv of freshwater is added from other sources (including runoff, mass loss of GrIS and $P - E$), such that 151 mSv of freshwater is transported by the EGC through Denmark Strait. For comparison, a recent study of Vage *et al.*[22] estimated the southward freshwater flux transported by the EGC through Denmark Strait is about 137 mSv, using a reference salinity of 34.80. Freshwater then continues southward in the EGC and the East Greenland Coastal Current (EGCC), an inner branch of the EGC. The EGC-EGCC system is shelf trapped with little freshwater lost offshore as it flows

southwards towards Cape Farewell, and receives added freshwater from Greenland mass loss, sea ice melt and $P - E$. Sutherland and Pickart[23] estimate 37 mSv (reference salinity is 34.80) of freshwater flux added to the EGC-EGCC system between 68 °N and Cape Farewell near 60 °N, based on measurements in 2004. The West Greenland Current (WGC) connects with the EGC-EGCC, transporting all of the freshwater that rounds Cape Farewell and any added freshwater from west Greenland northward, and into the Labrador Sea. Data in Myers *et al.*[24, 25] and Rykova *et al.*[26] as well as modeling studies[27, 28] show that virtually all of the freshwater that rounds Cape Farewell eventually ends up in the Labrador Sea. Thus, on the east side of Greenland, approximately 65% of Arctic Ocean freshwater exported through Fram Strait, and virtually all of the freshwater from Greenland that is added to the EGC-EGCC, is focused towards the Labrador Sea.

On the west side of Greenland, freshwater export from the Arctic Ocean (liquid freshwater plus sea ice) and freshwater from CAA glaciers and GrIS (melting plus calving) enters Baffin Bay, and then exits through Davis Strait in the Baffin Island Current[8, 29, 30]. This flows south into the Labrador Current, joined by freshwater outflow from Hudson Strait[31, 32]. All of this freshwater then flows south in the Labrador Current along the Labrador shelf. Myers[33] suggest that transport into the northern Labrador Sea interior here is relatively small, while Schmidt and Send[34] and McGeehan and Maslowski[35] suggest that some transport to the interior does occur here. The larger fraction of freshwater is exported farther south, around Flemish Cap and the Grand Banks[36-38], where much of it will be incorporated into the sub-polar gyre, eventually re-entering the EGC-EGCC and WGC, and ultimately, the Labrador Sea. Less than 25% of the freshwater passes south of the Grand Banks. Thus, at least 75% of the freshwater exported on the west side of Greenland from the three sources cited above (Arctic sea ice, GrIS and glaciers in the CAA) ultimately winds up in the Labrador Sea, either directly or indirecty through the sub-polar gyre.

With these various assumption and estimates, of the 20 mSv total increase of freshwater flux that we observe, at least 14 mSv (70%, of which 9 mSv is from GrIS and CAA and 5 mSv from Arctic sea ice), is advected into the Labrador Sea.

Since most of the 137 mSv of freshwater flux passing through Denmark Strait[22] on the east side of Greenland rounds Cape Farewell, and at least 75% of the 100 mSv of freshwater flux passing through Davis Strait[8] on the west side of Greenland eventually makes it to the Labrador Sea, total freshwater flux into the Labrador Sea likely exceeds 200 mSv. Thus, our estimate of increased freshwater flux into the Labrador Sea (14 – 20 mSv) may only represent 7 – 10% of the total. Future observations are required to refine these estimates and characterize their temporal variability.

Our study suggests that the sub-polar gyre's coastal currents focus increased freshwater from Greenland into the Labrador Sea, suppressing winter convection. Perhaps in the future the Nordic Seas will become more important relative to the Labrador Sea in terms of producing North Atlantic Deep Water and the southward return flow of the AMOC.

Supplementary References

1. Box, J. E. & Colgan, W. Greenland ice sheet mass balance reconstruction. part III: marine ice loss and total mass balance (1840–2010). *J. Clim.* **26,** 6990-7002 (2013).

2. Bamber, J., van den Broeke, M., Ettema, J., Lenaerts, J. & Rignot, E. Recent large increases in freshwater fluxes from Greenland into the North Atlantic. *Geophys. Res. Lett.* **39,** L19501 (2012).

3. Enderlin, E. M. *et al.* An improved mass budget for the Greenland ice sheet. *Geophys. Res. Lett.* **41,** 866–872 (2014).

4. Zhang, J. L. & Rothrock, D. A. Modeling global sea ice with a thickness and enthalpy distribution model in generalized curvilinear coordinates. *Mon. Weather Rev.* **131,** 845-861 (2003).

5. Good, S. A., Martin, M. J. & Rayner, N. A. EN4: Quality controlled ocean temperature and salinity profiles and monthly objective analyses with uncertainty estimates. *J. Geophys. Res. Oceans* **118,** 6704-6716 (2013).

6. Lenaerts, J. T. M. *et al.* Irreversible mass loss of Canadian Arctic Archipelago glaciers. *Geophys. Res. Lett.* **40,** 870-874 (2013).

7. Gardner, A. S. *et al.* Sharply increased mass loss from glaciers and ice caps in the Canadian Arctic Archipelago. *Nature* **473,** 357-360 (2011).

8. Haine, T. W. N. *et al*. Arctic freshwater export: status, mechanisms, and prospects. *Global Planet. Change* **125,** 13-35 (2015).

9. Aagaard, K. & Carmack, E. C. The Role of Sea Ice and Other Fresh-Water in the Arctic Circulation. *J. Geophys. Res. Oceans* **94,** 14485-14498 (1989).

10. Vavrus, S. J., Holland, M. M., Jahn, A., Bailey, D. A. & Blazey, B. A. Twenty-First-Century Arctic Climate Change in CCSM4. *J. Clim.* **25,** 2696-2710 (2012).

11. Proshutinsky, A., Dukhovskoy, D., Timmermans, M. L., Krishfield, R. & Bamber, J. L. Arctic circulation regimes. *Phil. Trans. R. Soc. A* **373,** 20140160 (2015).

12. Fetterer, F., Knowles, K., Meier, W. & Savoie, M. Sea Ice Index. National Snow and Ice Data Center, Boulder, Colorado (2002, updated daily).

13. Cavalieri, D. J., Parkinson, C. L. & Vinnikov, K. Y. 30-Year satellite record reveals contrasting Arctic and Antarctic decadal sea ice variability. *Geophys. Res. Lett.* **30,** 1970 (2003).

14. Kwok, R. *et al*. Thinning and volume loss of the Arctic Ocean sea ice cover: 2003–2008. *J. Geophys. Res.* **114,** C07005 (2009).

15. Maslowski, W., Kinney, J. C., Higgins, M. & Roberts, A. The future of Arctic sea ice. *Annu. Rev. Earth Pl. Sci.* **40,** 625-654 (2012).

16. Comiso, J. C., Parkinson, C. L., Gersten, R. & Stock, L. Accelerated decline in the Arctic sea ice cover. *Geophys. Res. Lett.* **35,** L01703 (2008).

17. Durbin, J. & Koopman, S. J. *Time Series Analysis by State Space Methods* 2nd edn (Oxford University Press, 2012).

18. Laine, M., Latva-Pukkila, N. & Kyrola, E. Analysing time-varying trends in stratospheric ozone time series using the state space approach. *Atmos. Chem. Phys.* **14,** 9707-9725 (2014).

19. Peterson, B. J. *et al.* Trajectory shifts in the Arctic and subarctic freshwater cycle. *Science* **313,** 1061-1066 (2006).

20. Bacon, S., Aksenov, Y., Fawcett, S. & Madec, G. Arctic mass, freshwater and heat fluxes: methods and modelled seasonal variability. *Phil. Trans. R. Soc. A* **373,** 20140169 (2015).

21. Dickson, R. *et al.* Current estimates of freshwater flux through Arctic and subarctic seas. *Prog. Oceanogr.* **73,** 210-230 (2007).

22. Vage, K. *et al.* Revised circulation scheme north of the Denmark Strait. *Deep Sea Res. Part I* **79,** 20-39 (2013).

23. Sutherland, D. A. & Pickart, R. S. The East Greenland Coastal Current: Structure, variability, and forcing. *Prog. Oceanogr.* **78,** 58-77 (2008).

24. Myers, P. G., Josey, S. A., Wheler, B. & Kulan, N. Interdecadal variability in Labrador Sea precipitation minus evaporation and salinity. *Prog. Oceanogr.* **73,** 341-357 (2007).

25. Myers, P. G., Donnelly, C. & Ribergaard, M. H. Structure and variability of the West Greenland Current in Summer derived from 6 repeat standard sections. *Prog. Oceanogr.* **80,** 93-112 (2009).

26. Rykova, T., Straneo, F. & Bower, A. S. Seasonal and interannual variability of the West Greenland Current System in the Labrador Sea in 1993-2008. *J. Geophys. Res. Oceans* **120,** 1318-1332 (2015).

27. Kawasaki, T. & Hasumi, H. Effect of freshwater from the West Greenland Current on the winter deep convection in the Labrador Sea. *Ocean Model* **75,** 51-64 (2014).

28. Saenko, O. A. *et al.* Role of Resolved and Parameterized Eddies in the Labrador Sea Balance of Heat and Buoyancy. *J. Phys. Oceanogr.* **44,** 3008-3032 (2014).

29. Curry, B., Lee, C. M. & Petrie, B. Volume, freshwater, and heat fluxes through Davis Strait, 2004-05. *J. Phys. Oceanogr.* **41,** 429-436 (2011).

30. Curry, B., Lee, C. M., Petrie, B., Moritz, R. E. & Kwok, R. Multiyear Volume, Liquid Freshwater, and Sea Ice Transports through Davis Strait, 2004-10*. *J. Phys. Oceanogr.* **44,** 1244-1266 (2014).

31. Straneo, F. & Saucier, F. The outflow from Hudson Strait and its contribution to the Labrador Current. *Deep Sea Res. Part I* **55,** 926-946 (2008).

32. St-Laurent, P., Straneo, F., Dumais, J. F. & Barber, D. G. What is the fate of the river waters of Hudson Bay? *J. Marine Syst.* **88,** 352-361 (2011).

33. Myers, P. G. Impact of freshwater from the Canadian Arctic Archipelago on Labrador Sea Water formation. *Geophys. Res. Lett.* **32,** L06605 (2005).

34. Schmidt, S. & Send, U. Origin and composition of seasonal Labrador sea freshwater. *J. Phys. Oceanogr.* **37,** 1445-1454 (2007).

35. McGeehan, T. & Maslowski, W. Impact of Shelf Basin Freshwater Transport on Deep Convection in the Western Labrador Sea. *J. Phys. Oceanogr.* **41,** 2187-2210 (2011).

36. Loder, J. W., Petrie, B. & Gawarkiewicz, G. The coastal ocean off northeastern North America: A large-scale view. *The Sea* **11,** 105-133 (1998).

37. Fratantoni, P. S. & Pickart, R. S. The western North Atlantic shelfbreak current system in summer. *J. Phys. Oceanogr.* **37,** 2509-2533 (2007).

38. Fratantoni, P. S. & McCartney, M. S. Freshwater export from the Labrador Current to the North Atlantic Current at the Tail of the Grand Banks of Newfoundland. *Deep Sea Res. Part I* **57,** 258-283 (2010).
